# Serine hydroxymethyl transferase is required for optic lobe neuroepithelia development in *Drosophila*

**DOI:** 10.1242/dev.201152

**Published:** 2023-06-01

**Authors:** Eunice A. B. Silva, Ana M. Venda, Catarina C. F. Homem

**Affiliations:** iNOVA4Health, NOVA Medical School, Faculdade de Ciências Médicas, NMS, FCM, Universidade Nova de Lisboa, 1169-056 Lisboa, Portugal

**Keywords:** One-carbon metabolism, Shmt, Neuroepithelia, *Drosophila*, Optic lobe

## Abstract

Cell fate and growth require one-carbon units for the biosynthesis of nucleotides, methylation reactions and redox homeostasis, provided by one-carbon metabolism. Consistently, defects in one-carbon metabolism lead to severe developmental defects, such as neural tube defects. However, the role of this pathway during brain development and in neural stem cell regulation is poorly understood. To better understand the role of one carbon metabolism we focused on the enzyme Serine hydroxymethyl transferase (Shmt), a key factor in the one-carbon cycle, during *Drosophila* brain development. We show that, although loss of Shmt does not cause obvious defects in the central brain, it leads to severe phenotypes in the optic lobe. The *shmt* mutants have smaller optic lobe neuroepithelia, partly justified by increased apoptosis. In addition, *shmt* mutant neuroepithelia have morphological defects, failing to form a lamina furrow, which likely explains the observed absence of lamina neurons. These findings show that one-carbon metabolism is crucial for the normal development of neuroepithelia, and consequently for the generation of neural progenitor cells and neurons. These results propose a mechanistic role for one-carbon during brain development.

## Teaser

**Summary:** Shmt, a key player in one-carbon metabolism, is essential for optic lobe development, and its loss leads to neuroepithelia apoptosis and morphological defects with consequent loss of neuroblasts and neurons.

## INTRODUCTION

Several lines of evidence support the crucial role of metabolism in the determination of cellular identity and in animal development. Metabolism consists of a complex system of metabolic pathways that can control cellular physiology by regulating energy production and the generation of substrates for biosynthetic pathways. Whereas studies of metabolic pathways regulating cell fate have been historically more focused on glycolysis and oxidative phosphorylation, in the past decades another metabolic pathway has been gaining attention: the one-carbon metabolism. This pathway has been connected to the process of embryonic development, cancer formation and neurodegenerative diseases (Locasale, 2013; Mattson and Shea, 2003; Stover, 2009; Xie et al., 2016).

One-carbon metabolism consists of a series of interconnecting metabolic pathways that function to transfer carbon units to acceptor substrates. The metabolic pathways of one-carbon metabolism are the folate cycle, the methionine cycle and the transsulfuration pathway (Appling, 1991; Clare et al., 2019; Tibbetts and Appling, 2010). Together, this network controls the synthesis of nucleotides, biosynthesis of lipids, the cellular redox status and methylation reactions by originating the universal methyl donor, S-adenosyl methionine (SAM). The one-carbon metabolism-dependent vitamin, folate (B9), is essential for normal development, and its imbalance during gestation and early childhood is linked to several developmental disorders, including neural tube defects (Engelhardt et al., 2022). Although the importance of folate and one-carbon metabolism in neural tube closure is unambiguous, the underlying mechanisms by which this occurs have not yet been fully dissected.

Serine hydroxymethyl transferase (Shmt) has been identified as a key enzyme of one-carbon metabolism, and thus a potential regulatory point of this metabolic pathway. Shmt catalyses the reversible reaction from serine to glycine and tetrahydrofolate (THF) to 5,10-methylene THF (meTHF), thus originating one-carbon units necessary for several metabolic reactions in the cell, including the synthesis of nucleotides and generation of universal methyl donor SAM for methylation reactions. Shmt has been shown to have a role in cellular growth and proliferation in mammals, *Caenorhabditis elegans* and *Drosophila* (Kim et al., 2015; Konrad et al., 2018; Liu et al., 2019; Winkler et al., 2017). In *C. elegans*, disruption of the *shmt* homologue causes an increase in cell cycle length (Liu et al., 2019). In *Drosophila*, lack of *shmt* causes embryonic cell cycle arrest, a phenotype rescued by the addition of dTTP nucleotides, suggestive of a possible role of *shmt* related to nucleotide production (Liu et al., 2019). One carbon metabolism has also been implicated in chromatin regulation, mainly by regulating histone methylation and phosphorylation (Liu et al., 2019; Li et al., 2015).

**Fig. 1.**
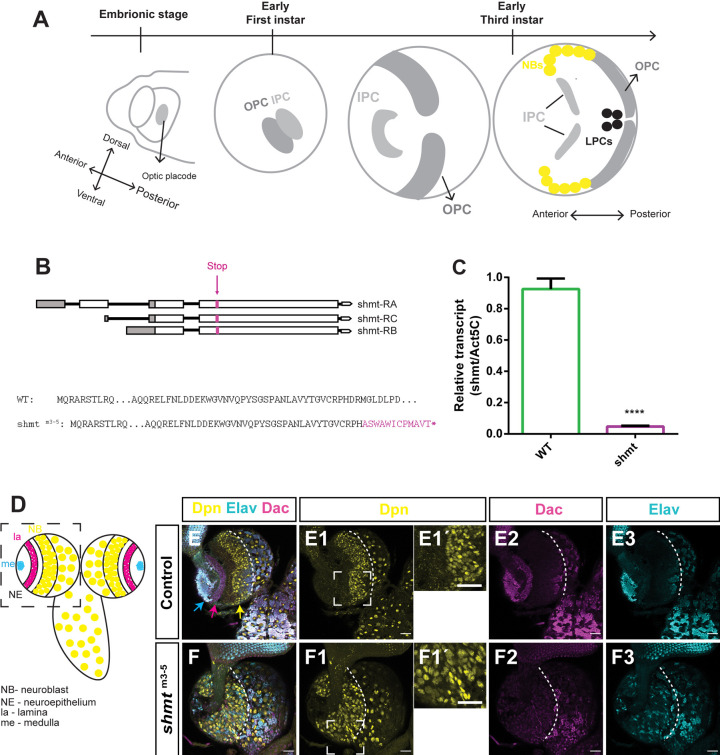
**Loss of *shmt* leads to defects in the larval optic lobe.** (A) Scheme of optic lobe (OL) development. In late embryonic stage, the OL neuroepithelium (NE) forms the optic placode. In early 1st instar larva, the NE splits into the outer proliferation centre (OPC) and the inner proliferation centre (IPC). In 3rd instar stages, these structures develop and generate medulla neuroblasts (NBs) and lamina progenitor cells (LPCs). (B) Scheme of *shmt*^m3-5^ mutation for the three isoforms. Amino acid sequence of wild type (WT) Shmt protein and *shmt*^m3-5^ mutant protein, which includes the frameshift and premature stop codon (asterisk) represented in magenta. (C) Quantification of mRNA levels of *shmt* in WT and in *shmt*^m3-5^ hemizygous larva by qPCR. Ratios of relative transcripts versus internal control gene, *act5C*. The error bars represent the standard deviation. ****P<0.0001, unpaired two-tailed Student's *t*-test. (D) Schematic of WT 3rd instar larval OL. The black dashed outline highlights one brain lobe. NBs in yellow, lamina (la) neurons in magenta and medulla (me) neurons in cyan (top view of the brain – anterior side). (E-F3) Control and *shmt*^m3-5^ wandering 3rd instar larval brains stained with anti-Deadpan (Dpn, yellow) to visualise NBs, anti-Elav to visualise medulla neurons (Elav, Cyan) and anti-Dachshund (Dac, magenta) to visualise lamina neurons. Dashed outline separates OL from central brain. Yellow arrow points medulla NBs, magenta arrow points lamina neurons, cyan arrow points medulla neurons. (E1′) Crop of control OL NBs. (F1′) Crop of *shmt*^m3-5^ OL NBs. w1118 was used as control. Scale bars: 20 μm.

The mechanistic role of one-carbon metabolism becomes particularly relevant in the context of brain development, where defects in this pathway are known to be particularly detrimental (Engelhardt et al., 2022). It is thus important to understand the role of *shmt* in neuroepithelia and in neural stem cell (NSC) fate regulation. To understand the role of one-carbon metabolism and Shmt in particular, we have analysed their role during brain development, focusing, in particular, in the optic lobe. The *Drosophila* optic lobe is a part of the brain responsible for adult visual processing and the formation of visual neurons, which count as ∼60% of total brain neurons (Nériec and Desplan, 2016). The process of optic lobe formation in *Drosophila* is very similar to mammalian cerebral cortex development (Brand and Livesey, 2011; Egger et al., 2011; Noctor et al., 2004). In the vertebrate cortex, neuroepithelial cells first divide symmetrically to expand the progenitor pool and then begin to differentiate into radial glial cells, which undergo asymmetric cell divisions to generate neurons and glial cells. Similarly, the *Drosophila* optic lobe develops from simple neuroepithelia, which first divide symmetrically to expand their progenitor population and then differentiate into NSCs (called neuroblasts in *Drosophila*; NBs). NBs then divide asymmetrically to generate ganglion mother cell (GMCs), which divide once more to form glia and neurons (Egger et al., 2007). Although neuroepithelial cells are formed in the embryo (Green et al., 1993), it is mainly during larval stages that the neuroepithelia expands and differentiates into postmitotic cells. The neuroepithelia starts as a single layer of epithelial cells, and during the 1st instar larval stage the neuroepithelia subdivide into two anlagen or ganglia: the inner and outer proliferation centre, the IPC and OPC, respectively (Fig. 1A) (Green et al., 1993). The IPC is responsible for the generation of precursor cells of the lobula complex neurons (divided into lobula and lobula plate) and the OPC forms the precursor cells of the medulla and lamina neurons (White and Kankel, 1978). The medulla is the major ganglia of the *Drosophila* larval optic lobe, originating ∼40,000 neurons (Chen and Desplan, 2020).

Here, we show that *shmt* is important for larval optic lobe development, with the loss of *shmt* leading to morphologically abnormal optic lobes, with smaller and defective neuroepithelia in the OPC. As knockdown or knockout (KO) of *shmt* specifically in the neuroepithelia fully recapitulates the optic lobe phenotype of animals mutant for *shmt*, we identify this tissue as the origin of this phenotype. Increased levels of apoptosis in the neuroepithelia partly explains their reduced size and the reduction in the number of NBs and medulla neurons. In addition, *shmt* mutant neuroepithelia do not form a lamina furrow, a structure essential for the formation of lamina neurons. Consistently, in *shmt*-depleted optic lobes there are no lamina neurons.

Interestingly, while a brain fully mutant for *shmt* is still capable of originating neuroepithelia and NBs, although abnormal, a single or small group of neuroepithelial cells mutant for *shmt*, surrounded by wild-type neuroepithelial cells, is very rare and, when present, grow poorly. Interestingly, in other epithelial tissues such as the wing disc, *shmt* mutant clones are also rare and the ones formed fail to grow. These results suggest a common mechanism in epithelia where lower levels of Shmt causes a growth disadvantage and epithelial cell elimination.

## RESULTS

### Loss of *shmt* leads to defects in the larval optic lobe

Humans and mice have two SHMT genes, but *Drosophila* has a single *shmt* gene located in the X chromosome (Winkler et al., 2017), facilitating its study. To investigate the role of *shmt* in brain and optic lobe development, we generated a *shmt* mutant fly (*shmt*^m3-5^) using the CRISPR-Cas9 technique (see Materials and Methods). *shmt*^m3-5^ is a 4 nucleotide deletion on exon 3 and is predicted to cause a frameshift from amino acid 120 that introduces a premature stop codon at amino acid 132, affecting all *shmt* isoforms (Fig. 1B)*.* The mutation in *shmt*^m3-5^ is thus upstream of the enzyme conserved active site (Garrow et al., 1993), which is predicted to start at amino acid 237.

As mutant male animals do not survive to adulthood it is not possible to obtain homozygous mutant females from the stock *shmt*^m3-5^/Fm7ActGFP (Fig. S1A,B) – for this reason all the following analysis were carried out with hemizygous mutant males. A viability analysis of *shmt*^m3-5^ animals revealed that males with the *shmt*^m3-5^ mutation (hemizygous animals) die in early pupal stages (Fig. S1B). To test whether this mutation leads to a reduction of *shmt* mRNA levels, we performed a quantitative reverse-transcription polymerase chain reaction (qRT-PCR) analysis, confirming a significant decrease of mRNA levels of *shmt* in *shmt*^m3-5^ compared with wild-type (WT) (Fig. 1C). The longer survival of zygotic *shmt*^m3-5^ animals in comparison with *shmt^X238^* maternal and zygotic mutants, which die in early stages of embryogenesis (Winkler et al., 2017), is likely due to maternal protein perdurance. This is supported by the presence of some Shmt protein, albeit in reduced levels, in the neuroepithelia in late L2 stages (Fig. S1C,D).

To better explore the role of *shmt* in brain and optic lobe development, and as *shmt*^m3-5^ hemizygous animals die at pupal stages, we started by analysing animals in the 3rd instar larval stage, the stage that precedes pupal formation. We dissected wandering 3rd instar larval brains and analysed their optic lobes using an anti-Deadpan (Dpn) antibody to mark optic lobe NBs, an anti-Elav antibody to identify medulla neurons and an anti-Dachshund (Dac) to identify lamina neurons (Fig. 1D). This analysis revealed that *shmt*^m3-5^ hemizygous mutant brains had abnormal optic lobes (Fig. 1E,F), with absent lamina and reduced number of medulla neurons in ectopic locations (Fig. 1E2,E3,F2,F3). Furthermore, the optic lobe NBs were disorganised (Fig. 1F) and were larger than in control optic lobes [control optic lobe NB diameter=4.18±0.08 µm (*n*=68); *shmt*^m3-5^ optic lobe NB diameter=6.49±0.13 µm (*n*=67; *P*<0.0001, unpaired two-tailed Student's *t*-test) (Fig. 1E1-E1′,F1-F1′)]. The disorganised optic lobe phenotype observed in *shmt*^m3-5^ was nicely rescued by the introduction of a genomic rescue construct that includes WT *shmt (shmt+)* (Winkler et al., 2017) (Fig. S1E-G). This confirms that the observed phenotypes are specifically caused by the mutation in *shmt*.

In contrast, the central brain of *shmt*^m3-5^ animals had no obvious defects (Fig. S2A,B). In the central brain, NB lineages can be subdivided according to their lineages into Type I (∼100 per lobe) and Type II (eight per lobe). In *shmt*^m3-5^ brains the number and size of type I and type II NBs was similar to the control situation (Fig. S2C-F). The mitotic rates observed in *shmt*^m3-5^ central brains were also unchanged compared with the control (Fig. S2G). A more detailed analysis of type I lineages, which represent the large majority of lineages in the central brain, revealed that there was no change in the number of NBs per lineage (Fig. S2H) although there was a slight change in the distribution of the number of GMCs per lineage (Fig. S2I). The number of GMCs per lineage normally varies between two and four, averaging at three. Lineages in *shmt*^m3-5^ mutants also had two to four GMCs, averaging at three, but there was a higher percentage of lineages that had only two GMCs and a lower percentage of lineages that had four GMCs.

Taken together, these results indicate that *shmt* is required for proper brain development affecting primarily optic lobe development.

### Loss of *shmt* in the neuroepithelia leads to defects in the larval optic lobe

**Fig. 2.**
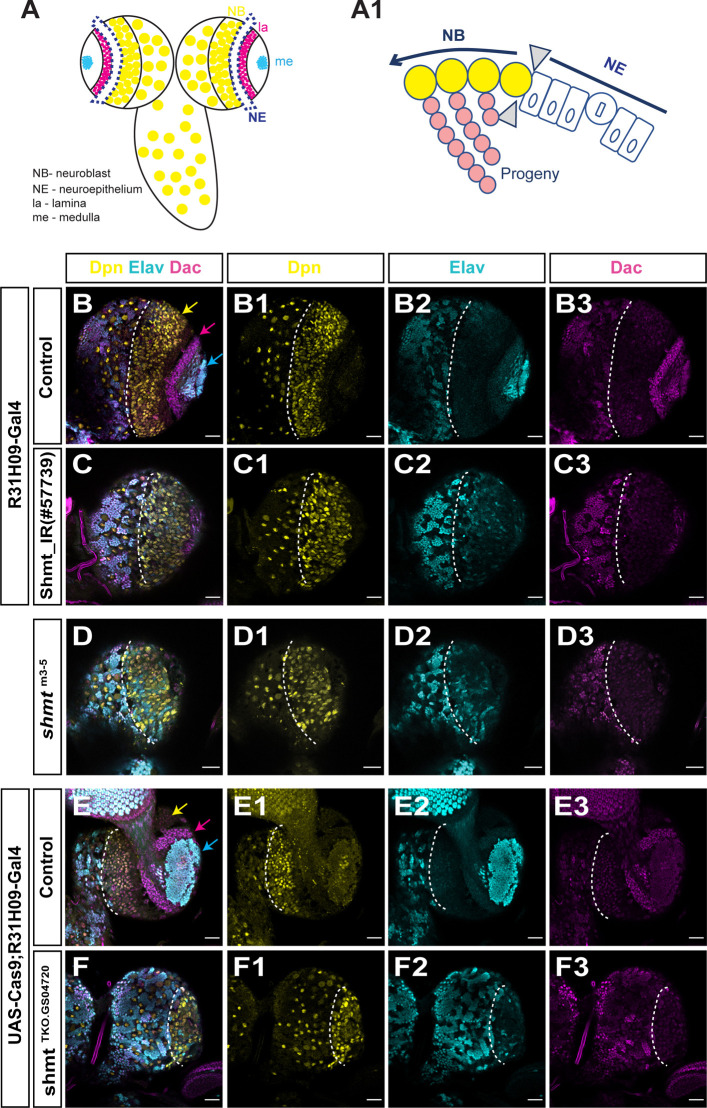
**Loss of *shmt* in the neuroepithelia leads to defects in larval optic lobe.** (A) Schematic of WT optic lobe (OL) (top view of the brain – anterior side). Blue dashed lines show outer proliferation centre (OPC) neuroepithelia (NE). (A1) Scheme of OPC neuroepithelia transition to neuroblast (NBs) in WT larval OL. (B-F3) Wandering 3rd instar larval brains of the indicated genotypes stained with anti-Deadpan (Dpn, yellow) to visualise NBs, anti-Dachshund (Dac, magenta) to visualise lamina and anti-Elav (Elav, cyan) to visualise neurons. Dashed outline separates OL from central brain. (B,E) Control brain lobes: control used was w1118 crossed with the respective Gal4 driver. Yellow arrow points to medulla NBs, magenta arrow points to lamina neurons, cyan arrow points to medulla neurons. (C-C3) *shmt* RNAi in neuroepithelium (NE), driven by R31H09-Gal4. (D-D3) *shmt*^m3-5^ hemizygous mutant brain. (F-F3) *shmt* KO in NE induced by UAS-Cas9;R31H09-Gal4 and *Shmt^TKO.GS04720^*. Scale bars: 20 µm.

To understand the mechanisms that lead to a defective optic lobe in *shmt*^m3-5^ animals, we first set out to identify what is the cellular origin of the observed optic lobe phenotype. The majority of NBs in the optic lobe are originated from symmetrically dividing OPC neuroepithelial cells during larval stages (Egger et al., 2007) (Fig. 2A-A1). As in *shmt* mutant brains there were no lamina neurons and fewer medulla neurons (both originated by OPC neuroepithelial cells), we hypothesised that the loss of *shmt* could lead to an impairment in the transition of OPC neuroepithelial cells to precursor cells.

To test this hypothesis, we knocked down *shmt* using RNA interference (RNAi; Shmt_IR) specifically in the neuroepithelia, using an embryonic neuroepithelia driver, R31H09-GAL4 (Hakes et al., 2018), which is also expressed in the larval neuroepithelia (Fig. S3). RNAi knockdown of *shmt* in the neuroepithelia resulted in a phenotype that largely mimics the phenotype of *shmt*^m3-5^ mutant animals, causing disorganised optic lobes with almost no lamina or medulla neurons (Fig. 2B-C3). However, in brains where *shmt* was knocked down only in the neuroepithelia, the phenotype was less severe than in the full mutant, as it was still possible to visualise an underdeveloped lamina that was completely absent in *shmt*^m3-5^ optic lobes (Fig. 2D-D3). This small difference in the phenotype severity between the *shmt* RNAi and the full mutant may be due to the different levels of remaining Shmt activity in each situation. To test this, we knocked-out *Shmt* specifically in neuroepithelia cells using tissue-specific CRISPR-Cas9 (Lin et al., 2015; Ren et al., 2013). To induce mutagenesis of *Shmt* in the neuroepithelia, we used a UAS-Cas9 transgene and the R31H09-GAL4 driver in the presence of a ubiquitously expressed small guide RNA (sgRNA) for *shmt* (*Shmt^TKO.GS04720^*). Neuroepithelia-specific KO of *shmt* led to a reduced number of medulla neurons and to an absence of lamina neurons (Fig. 2E-F3), as previously observed in *shmt*^m3-5^ animals (Fig. 2D-D3). These results show that the ablation of *shmt* solely in neuroepithelia cells recapitulates the optic lobe defect seen in animals completely mutant for *shmt*. This suggests that *shmt* is important for neuroepithelia development and indicates that the neuroepithelia cells are at the root of the observed optic lobe phenotype.

### Downregulation of Thymidylate synthase leads to defective larval optic lobes

**Fig. 3.**
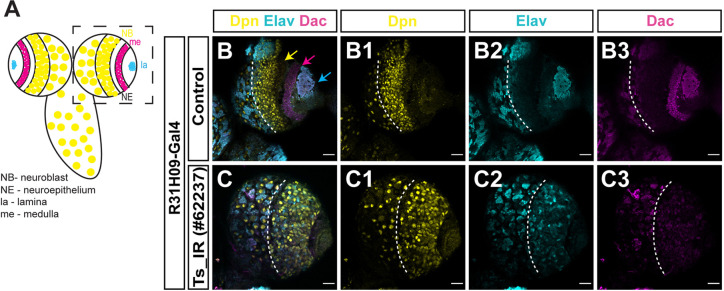
**Downregulation of Thymidylate synthase leads to defective larval optic lobes.** (A) Schematic of WT larval optic lobe (OL) (top view of the brain – anterior side). Dashed outline shows one brain lobe. (B-C3) Wandering 3rd instar larval brains of control (w1118) and Thymidylate synthase (*Ts*) RNAi, crossed to a neuroepithelium driver (R31H09-Gal4), with anti-Deadpan (Dpn, yellow) to visualise neuroblasts (NBs), anti-Dachshund (Dac, magenta) to visualise lamina and anti-Elav (Elav, cyan) to visualise neurons. Yellow arrow points to medulla NBs, magenta arrow points to lamina neurons, cyan arrow points to medulla neurons. Dashed lines separate OL from central brain. Scale bars: 20 µm.

To test whether the observed defective optic lobe phenotype is specifically related to *shmt* or whether it is a consequence of a defective one-carbon cycle, we decided to knockdown another enzyme from the one-carbon cycle to determine if the cycle is globally required for neuroepithelia and optic lobe development. Knockdown of Thymidylate synthase (Ts; folate cycle enzyme) in the neuroepithelia using Ts RNAi (Ts_IR) and the neuroepithelia driver R31H09-GAL4 led to an absence of lamina neurons and to a reduced number of medulla neurons, abnormally located (Fig. 3A-C); a phenotype similar to that observed in *shmt* mutants (Fig. 2D). These results indicate that optic lobe development depends on correct activity of the one-carbon cycle, and not on the function of Shmt specifically.

### Loss of *shmt* leads to smaller neuroepithelia, failure in lamina furrow formation and a reduced number of neuroblasts formed

**Fig. 4.**
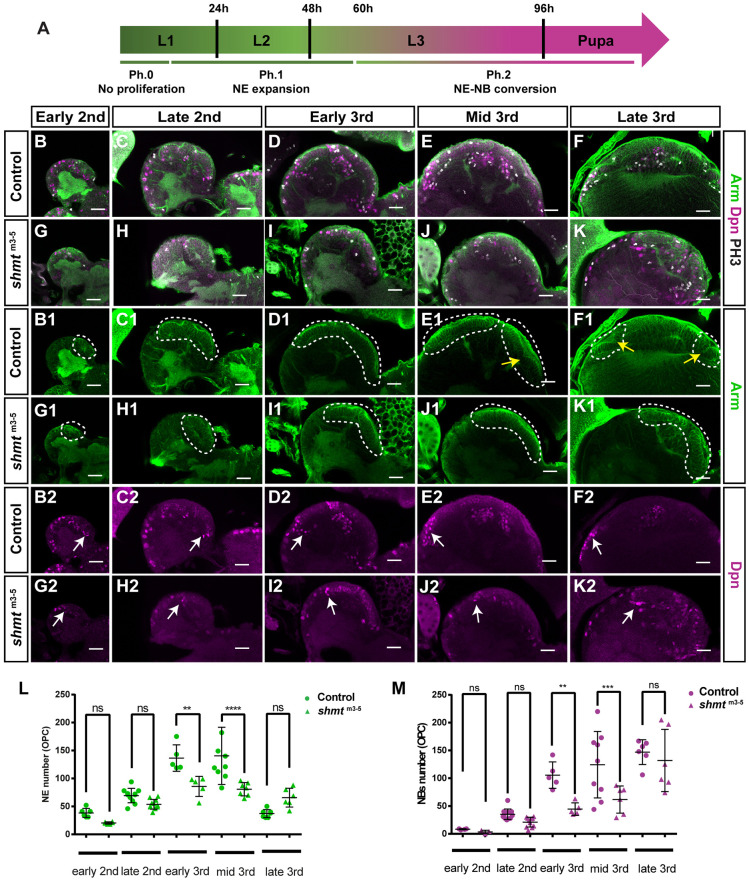
**Loss of *shmt* leads to smaller neuroepithelia, failure in lamina furrow formation and reduced number of neuroblasts formed.** (A) Schematic of the different phases of neuroepithelia progression. In Phase 0 (Ph.0) there is little to no proliferation of neuroepithelial cells (NE). In Phase 1 (Ph.1) there is mostly NE expansion. In Phase 2 (Ph.2), neuroepithelia-to-neuroblast (NE-NB) conversion results in a reduction of NE population. (B-K2) Neuroepithelial development of control and *shmt*^m3-5^ at the indicated larval stages. Larval brains were stained with anti-Armadillo (Arm, green) to visualise neuroepithelia, with anti-Deadpan (Dpn, magenta) to visualise neuroblasts (NBs) and anti-Phospho-histone H3 (PH3, grey) to visualise dividing cells. Dashed lines mark neuroepithelial region. Yellow arrows show the lamina furrow. White arrows point to neuroblasts in the outer proliferation centre (OPC) region. (L) Quantification of OPC neuroepithelia cell number for control and *shmt*^m3-5^ during larval neuroepithelial development. (M) Quantification of OPC NB number for control and *shmt*^m3-5^ during larval development. w1118 was used as control. Number of brains quantified for control: early 2nd (24 h ALH, *n*=6), late 2nd (48 h ALH, *n*=12), early 3rd (60 h ALH, *n*=5), mid 3rd (72 h ALH, *n*=9), late 3rd (96 h ALH, *n*=7). Number of brains quantified for *shmt*^m3-5^: early 2nd (*n*=6), late 2nd (*n*=11), early 3rd (*n*=5), mid 3rd (*n*=7), late 3rd (*n*=6). Error bars represent the standard deviation. **P<0.01, ***P<0.001, ****P<0.0001 (Bonferroni's multiple comparison test). ns, not significant (*P*>0.05). Scale bars: 20 µm.

Our results suggest that neuroepithelial cells are at the origin of the optic lobe phenotype observed in *shmt* mutants. The developing OPC of the optic lobe arises in the L1 larval stage from a small group of progenitor cells, which form an epithelial sheet that first expands and then converts into NBs. This process can be divided into three phases (Fig. 4A): Phase zero (Ph.0), occurs during the L1 larval stage, in which there is little or no proliferation of neuroepithelial cells; Phase one (Ph.1), characterised mostly by neuroepithelia expansion, occurs in the L2 larval stage, during which neuroepithelial cells divide symmetrically and expand their population; and Phase two (Ph.2), characterised mostly by neuroepithelia-NB conversion at the transition zone, occurs in the L3 larval stage, when a wave of proneural gene expression gradually converts neuroepithelial cells to asymmetrically dividing NBs (Egger et al., 2007; Yasugi et al., 2008).

To understand how deficient levels of Shmt affect the OPC neuroepithelia, we analysed neuroepithelia development throughout larval stages in *shmt* mutants (neuroepithelia quantification method in Fig. S4A and in Materials and Methods). At early 2nd stages, the neuroepithelia of *shmt*^m3-5^ animals were not significantly different from controls (Fig. 4B,G,L). At late 2nd stage, the neuroepithelia in *shmt*^m3-5^ appeared to be morphologically different from control, but the number of neuroepithelial cells in these animals was not significantly different from controls (Fig. 4C,H,L). At early 3rd and mid 3rd larval stages the neuroepithelia tissue in *shmt*^m3-5^ animals was significantly smaller (Fig. 4D,E,I,J,L). Interestingly, *shmt*^m3-5^ animals did not have a visible lamina furrow at mid 3rd stages, when it is normally present in the control situation (Fig. 4E1,J1, yellow arrow shows the lamina furrow), nor did they form it later in development (Fig. 4F1,K1). As the lamina furrow is essential for lamina neurogenesis, the absence of this structure could explain why *shmt*^m3-5^ animals do not form lamina neurons (Fig. 1F). This failure to convert neuroepithelia into lamina precursor cells (LPCs) was consistent with a relative increase in neuroepithelial cells observed in *shmt*^m3-5^ mutant animals in late 3rd larval stage (Fig. 4L), compared with same stage controls where neuroepithelial cells had converted into LPCs/neurons. These remaining neuroepithelial cells did, however, have a defective shape, being rounder and larger (Fig. 4K; larger view in Fig. S4B,C). As the neuroepithelia in *shmt* mutant animals in mid 3rd and late 3rd stages are similar in size to late 2nd control animals, which are 24 h and 36 h younger, respectively, we have tested whether *shmt* mutant animals are developmentally delayed. We have quantified the developmental time from embryo until pupariation and found that *shmt* mutant animals suffer only an 8 h delay [control 121±0.77 h (*n*=340), *shmt*^m3-5^ 113±1.35 h (*n*=118)]. So, the neuroepithelia smaller size cannot be solely explained by a delay in animal development. In addition, this small delay does not explain the morphological neuroepithelia defects observed, such as the absence of lamina furrow.

In *shmt* mutant animals, we observed fewer medulla neurons that, in addition, were spread in ectopic locations (Fig. 1F). We thus hypothesised that the defects observed in neuroepithelia could be affecting the number of medulla NBs formed in *shmt* mutants. We have thus quantified the number of NBs (anti-Dpn-positive cells) in optic lobes throughout larval stages, and consistently found significantly fewer NBs in *shmt*^m3-5^ optic lobes compared with control in early 3rd and mid 3rd instar larval stages (Fig. 4M).

### *shmt* mutant neuroepithelia have increased levels of apoptosis

**Fig. 5.**
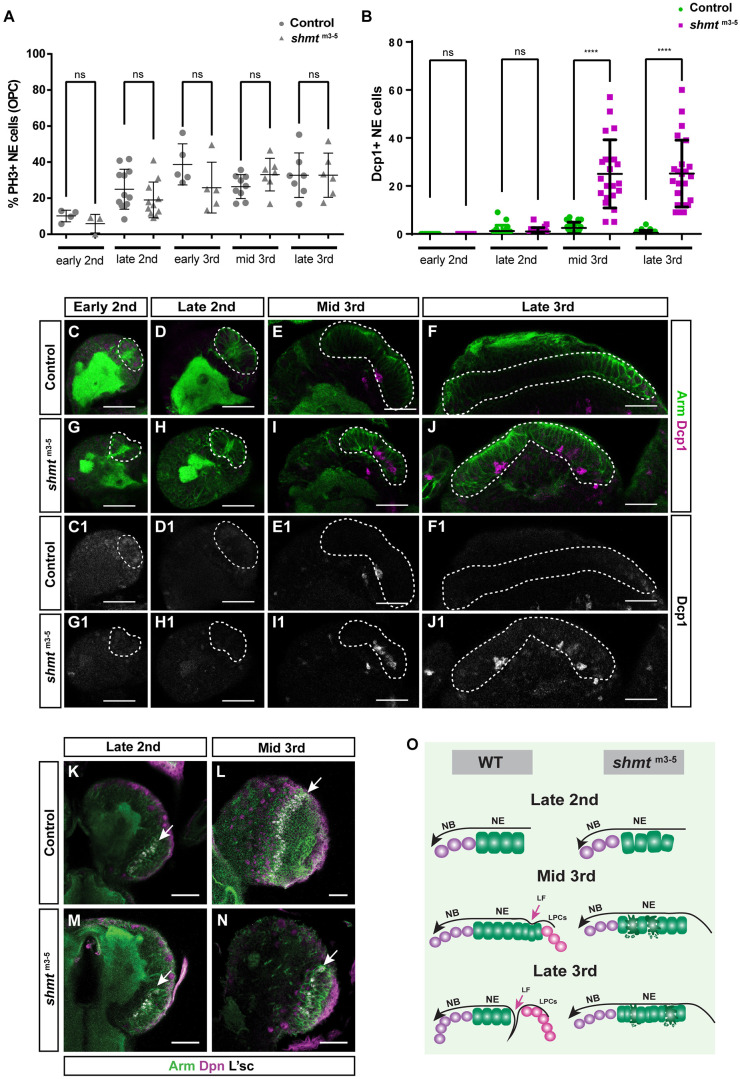
**Loss of *shmt* leads to increased apoptosis in the neuroepithelia.** (A) Quantification of Phospho-histone H3-positive (PH3+) neuroepithelial (NE) cells for control and *shmt*^m3-5^ during larval development. Number of brains quantified for control: early 2nd (24 h ALH, *n*=6), late 2nd (48 h ALH, *n*=12), early 3rd (60 h ALH, *n*=5), mid 3rd (72 h ALH, *n*=9), late 3rd (96 h ALH, *n*=7). Number of brains quantified for *shmt*^m3-5^: early 2nd (*n*=6), late 2nd (*n*=11), early 3rd (*n*=5), mid 3rd (*n*=7), late 3rd (*n*=6). (B) Quantification of number of *Drosophila* cleaved caspase-1-positive (Dcp1+) cells in total outer proliferation centre (OPC) NE through larval development, for control and *shmt*^m3-5^. Number of brains quantified for control: early 2nd (*n*=14), late 2nd (*n*=20), mid 3rd (*n*=23), late 3rd (*n*=23). Number of brains quantified for *shmt*^m3-5^: early 2nd (*n*=13), late 2nd (*n*=22), mid 3rd (*n*=21), late 3rd (*n*=23). The error bars represent the standard deviation. ****P<0.0001 (Bonferroni's multiple comparison test). ns, not significant. (C-J1) Neuroepithelia of control and *shmt*^m3-5^ at the indicated larval stages. Larval brains were stained with anti-Armadillo (Arm, green) to visualise NE and anti-Dcp1 (Dcp1, magenta or grey) to visualise apoptotic cells. Dashed lines mark neuroepithelial cells region. (K-N) Control and *shmt*^m3-5^ optic lobe (OL) of the indicated larval stage. Brains were stained with anti-Armadillo (Arm, green) to visualise NE, anti-Deadpan (Dpn, magenta) to visualise neuroblasts (NBs) and anti-L'sc (grey) to visualise the NE-NB transition zone. White arrows point to NE-NB transition zone. w1118 was used as control. (O) Model for the differentiation of neuroepithelia through larval OL development of WT and *shmt*^m3-5^. *shmt* mutants have smaller neuroepithelia from mid 3rd instar stages, accompanied by significantly higher levels of apoptosis in the NE, justifying the reduction in the size of this epithelium from this stage onward. At late 3rd instar, control OL have a reduction in the number of NE cells as they have mostly converted into NBs and lamina precursor cells (LPCs). *shmt* mutants fail to form a lamina furrow (LF) and convert neuroepithelia into LPCs. Scale bars: 20 µm.

To try to mechanistically understand why the neuroepithelia of *shmt*^m3-5^ animals do not grow as normal, we started by testing whether the mitotic rate of neuroepithelial cells or optic lobe NBs was altered using the mitotic marker PH3 (Fig. 5A). We have additionally used 5-ethynyl-2-deoxyuridine (EdU) labelling, which is incorporated into nascent DNA during S-phase (Fig. S4D). The quantifications of PH3- and EdU-positive neuroepithelia cells showed that there were no statistical differences between the mitotic rate between control and *shmt*^m3-5^ through all larval stages analysed (Fig. 5A; Fig. S4D). These results indicate that, even though there are fewer neuroepithelia cells in *shmt*^m3-5^ optic lobes, they are still mitotically active. Although the NB number was reduced in *shmt*^m3-5^ optic lobes, the existing NBs also had a normal mitotic rate (Fig. S4E).

We next tested whether some *shmt* mutant neuroepithelial cells might be dying. We analysed the neuroepithelia of *shmt* mutant animals for the presence of apoptotic cells using an established antibody against cleaved Dcp1, which reports Caspase 3 (Casp3) activity, marking apoptotic cells (Özel et al., 2021). Neuroepithelia in early 2nd and late 2nd stages had normal levels of apoptosis (Fig. 5B,C,D,G,H). However, from mid 3rd to late 3rd stages there was a significant increase in the number of apoptotic neuroepithelial cells (Fig. 5B,E,F,I,J). Interestingly, these results suggest that the increased levels of neuroepithelial cell death during 3rd instar stages, during the expansion phase and the neuroepithelium-NB conversion phase, likely contributes to the reduction in neuroepithelia size during these stages and consequently the reduction in the number of the progeny generated.

Although the reduced size of the neuroepithelia might by itself justify the reduced number of NBs formed in *shmt* mutant brains, additional defects in the neuroepithelium-NB fate transition could also contribute to the phenotype. A key factor to induce the neuroepithelia to NB transition is the proneural factor Lethal of scute (L'sc). We therefore next analysed whether the L'sc positive transition zone might be impaired in *shmt* mutants. Staining for L'sc with a specific antibody (Suzuki et al., 2013) revealed that L'sc was expressed as expected in a stripe in both late L2 and mid L3 stages, even though the morphology of the optic lobe was abnormal in *shmt* mutants (Fig. 5K-N).

In summary, these results indicate that the loss of *shmt* induces higher rates of neuroepithelial cell death during the expansion and neuroepithelium-NB conversion phases (Fig. 5O). As the mitotic rates of neuroepithelia are normal and the neuroepithelium-NB fate conversion is not impaired, the increased levels of apoptosis of neuroepithelia are likely responsible for the reduced number of neuroepithelial cells and NBs. How or whether the reduction in neuroepithelial cells contributes to the defects in lamina furrow formation, or if this occurs through an independent mechanism, remains unanswered.

### Unequal levels of *shmt* in neuroepithelial cells lead to cell growth disadvantage

**Fig. 6.**
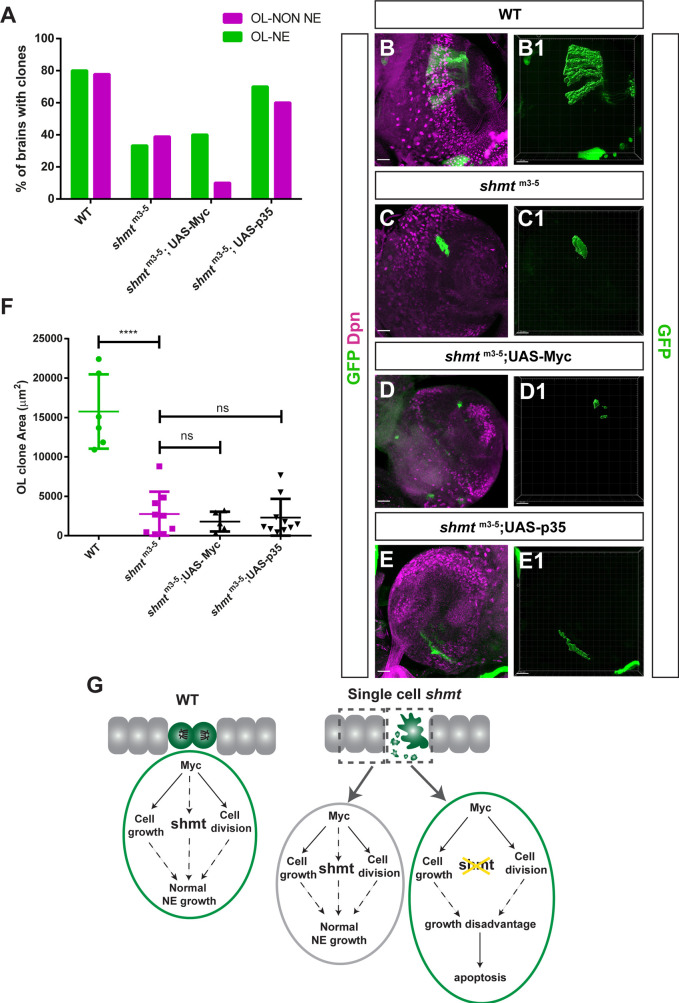
***shmt* neuroepithelial clones have a growth disadvantage.** (A-E) Optic lobe (OL) MARCM clones generated at 48 h ALH, analysed at wandering 3rd instar larval stage. (A) Quantification of brains with OL clones in neuroepithelia (OL NE) and brains with OL clones not localised in NE (OL-NON NE) of control, *shmt*^m3-5^, *shmt*^m3-5^;UAS-Myc and *shmt*^m3-5^;UAS-p35. Number of brains analysed: WT (*n*=10), *shmt*^m3-5^ (*n*=18), *shmt*^m3-5^;UAS-Myc (*n*=10), *shmt*^m3-5^;UAS-p35 (*n*=10). Statistical analysis with Fisher's exact test. OL-NE (clones present versus absent): [WT versus *shmt*^m3-5^, *P*<0.05]; [*shmt*^m3-5^ versus *shmt*^m3-5^;UAS-Myc, *P*=ns]; [*shmt*^m3-5^ versus *shmt*^m3-5^;UAS-p35, *P*=ns]. OL- NON-NE (clones present versus absent): [WT versus *shmt*^m3-5^, *P*=ns]; [*shmt*^m3-5^ versus *shmt*^m3-5^;UAS-Myc, *P*<0.05]; [*shmt*^m3-5^ versus *shmt*^m3-5^;UAS-p35, *P*=ns]. (B-E) Larval brains of indicated genotypes stained with anti-Deadpan (Dpn, magenta) to visualise NBs; clones marked by CD8::GFP (GFP, green). (B1-E1) Imaris 3D projection of GFP-positive clone volume for the indicated genotype. (F) Quantification of control and *shmt*^m3-5^ optic lobe MARCM clone area (OL NE+OL-NON NE) in wandering 3rd instar larvae. Number of clones: WT (*n*=6), *shmt*^m3-5^ (*n*=10), *shmt*^m3-5^;UAS-Myc (*n*=4), *shmt*^m3-5^;UAS-p35 (*n*=10). Error bars represent the standard deviation. ****P<0.0001 (Bonferroni multiple comparison test). For all experiments, FRT19A was used as control. (G) Model for neuroepithelial clone growth and survival signalling pathway. Purple cells represent WT neuroepithelial cells; green cells represent MARCM clones of indicated genotypes. ns, not significant. Scale bars: 20 µm.

To better understand how neuroepithelial cells depleted of *shmt* originate defective optic lobes, we next wanted to test whether single *shmt* mutant neuroepithelia cells originate the stereotypical lineage with appropriate number and cell fates. For this, we performed a mosaic clone lineage analysis and generated *shmt*^m3-5^ GFP-positive mosaic clones which would allow us to track and assess the fate of the progeny generated by these mutant neuroepithelial cells (MARCM; Lee and Luo, 2001). The *shmt*^m3-5^ clones were induced in 2nd [24 h after larval hatching (ALH); Fig. S5A,B] and early 3rd (48 h ALH; Fig. 6) instar larval stages, as these stages encompass the time windows in which neuroepithelia are expanding their number, thus increasing the chance of generating neuroepithelia clones. The mosaic brains were then analysed at wandering 3rd instar larval stage. To our surprise, after many attempts to induce clones in the neuroepithelia, we were only able to generate very few *shmt*^m3-5^ clones. We have quantified the frequency of WT and *shmt*^m3-5^ clone formation in both the neuroepithelia and in non-neuroepithelial optic lobe regions (clones present versus clones absent) and this analysis revealed that the number of *shmt*^m3-5^ clones in the optic lobe was significantly lower than the number of WT clones in both optic lobe regions (WT versus *shmt*^m3-5^, *P<0.05; WT versus *shmt*^m3-5^, ****P<0.0001; Fisher's exact test) (Fig. 6A; Fig. S5A). In addition to the reduced frequency of *shmt* mutant clones, the *shmt*^m3-5^ clones that were generated were significantly smaller compared with control (Fig. 6B,C,F, Fig. S5B). Taken together, these results indicate that the lack of *shmt* in an optic lobe clone leads to a disadvantage in clone formation.

As *shmt* clones were only generated for a maximum of 72 h, and as we have previously shown that the Shmt protein is quite stable (Fig. S1C), we expected *shmt*^m3-5^ mutant clones to have a weaker phenotype than *shmt* zygotic mutants. However, the rarity in *shmt*^m3-5^ clone formation and their low proliferation rates contrasts with *shmt* zygotic mutants, for which neuroepithelia are still formed and NBs and neurons generated, albeit in reduced numbers. The fact that the *shmt*^m3-5^ neuroepithelia clones are surrounded by WT cells led us to hypothesise that they were being subjected to ‘cell competition’. Cell competition occurs usually in rapidly proliferating epithelial tissues where less-fit cells with slower growth rate originate a smaller clone size which is gradually eliminated from the WT tissue, normally by apoptosis (Morata and Ripoll, 1975; Moreno et al., 2002; Moreno and Basler, 2004). It has been shown that higher expression of growth regulator Myc give cells a growth advantage relative to their neighbours (Moreno and Basler, 2004). Interestingly, in mammals Shmt was shown to act in the Myc pathway (Nikiforov et al., 2002).

To test whether single neuroepithelia cells mutant for *shmt* were being eliminated by cell competition, we gave *shmt* neuroepithelia cells an additional growth advantage by overexpressing the growth factor Myc (Gallant, 2013) with the intent of preventing their elimination and increasing the number of clones recovered. However, the overexpression of Myc did not increase the size or number of *shmt* neuroepithelia clones (Fig. 6A,D,F). This could suggest that Shmt may act downstream of Myc, as happens in mammals (Nikiforov et al., 2002). Another assay that is commonly used to test whether cell competition is involved in cell elimination is to block apoptosis, the final step in the mechanism by which less fit cells are normally eliminated (Moreno and Basler, 2004). We thus tested whether blocking apoptosis by overexpressing p35 (Moreno and Basler, 2004) in *shmt* mutant clones increased their survival. Increasing the levels of p35 in *shmt*^m3-5^ clones led to an increase in clone number, although not in a significant manner (*shmt*^m3-5^ versus *shmt*^m3-5^;UAS-p35: *P*=0.059, Fisher's exact test) (Fig. 6A). However, the area of these clones was not increased when compared with *shmt*^m3-5^-deficient clones only (Fig. 6E,F). This result is expected, as preventing the elimination of *shmt* mutant cells still does not rescue their defective one-carbon metabolism, which is responsible for originating important cellular building blocks, such as nucleotides. Thus, while the *shmt*^m3-5^ clones can partially survive if their apoptosis is prevented, they are still not fit enough to divide. To test whether higher levels of Shmt could alone be an advantage for cell growth we have generated clones overexpressing Shmt-HA, this however did not cause a growth advantage (Fig. S5C). This result is not unexpected, as the rate limiting step for most metabolic enzymes is the concentration of their substrate.

To test whether the observed requirement of *shmt* in the brain neuroepithelia is brain specific or whether its role is conserved in all epithelia, we decided to test whether *shmt* depletion in other epithelial tissues causes similar phenotypes. For this, we analysed *shmt*^m3-5^ clones in 3rd instar larval imaginal wing discs. Similar to what we observed in the optic lobe neuroepithelia, *shmt*^m3-5^ wing disc clones were also much smaller and rarer than WT clones (Fig. S5D,E). These results suggest that the growth disadvantage of *shmt*^m3-5^ clones is conserved in several epithelial tissues. Altogether, these results indicate that *shmt* is important for the growth of epithelial cell clones and that epithelial cells lacking *shmt* in a WT tissue have a growth disadvantage.

## DISCUSSION

Here, we show that *shmt* is important for proper optic lobe development during larval stages, in particular in the neuroepithelia. During the larval stages, more concretely in the 3rd instar larval stage, neuroepithelial cells proliferate rapidly to expand their population and form the medulla NBs and LPCs that will then originate the medulla and lamina neurons, respectively, which together will compose the adult visual system. Hence, during this developmental window, there is a high demand of cellular building blocks, such as nucleotides for DNA replication, which are necessary for correct cell division and proliferation. Shmt acts upstream of the nucleotide synthesis pathway, responsible for the conversion of serine to glycine and THF to meTHF. The meTHF formed is then used to originate the purine nucleotides by the enzyme Ts. In *Drosophila* embryos, it has been shown that *shmt* is important for nucleotide synthesis, and the loss of *shmt* leads to cell cycle arrest and decreased cell proliferation during this stage (Liu et al., 2019; Winkler et al., 2017). Our results show that the *shmt* mutant phenotype originates in the neuroepithelia. *shmt* mutants have a lower number of neuroepithelial cells from early 3rd instar larval stages. During 3rd instar stages, *shmt* mutant neuroepithelia have high levels of apoptosis (Fig. 5O), likely explaining the reduction in the size of this structure and in the number of NBs and neurons generated. These increased rates of apoptosis in *shmt* mutant neuroepithelia might be a response to less fit cells caused by insufficient levels of one-carbon cellular building blocks. The mitotic rates of the remaining neuroepithelia and NBs in *shmt* mutants are, however, not different when compared with WT. Combined, this suggests that there is heterogeneity in cellular response to the absence of *shmt*.

Consistently, when we generated *shmt* mutant neuroepithelial cell clones, we observed that heterogeneous levels of *shmt* in neighbouring cells is particularly detrimental, leading to a reduced number of *shmt* neuroepithelial cell mutant clones recovered. As *shmt* clones were only generated for a maximum of 72 h, and the Shmt protein is quite stable (Fig. S1C), we expected *shmt*^m3-5^ mutant clones to have a weaker phenotype than *shmt* zygotic mutants. However, the rarity in *shmt*^m3-5^ clone formation and their low proliferation rates contrasts with *shmt* zygotic mutants, in which neuroepithelia are still formed and NBs and neurons generated, albeit in reduced numbers. The fact that the *shmt*^m3-5^ neuroepithelia clones are surrounded by WT cells made us hypothesise that these Shmt-defective cells could be eliminated by a mechanism similar to cell-competition. Although it has been shown that cells with high levels of Myc compete more and grow more than their WT neighbours (Moreno and Basler, 2004), the overexpression of the growth factor Myc in *shmt*-deficient clones did not rescue clonal growth. A possible reason might be due to *shmt* acting downstream of Myc, as shown in rat fibroblasts (Nikiforov et al., 2002). Consistently, it has been shown that when the protein synthesis machinery is compromised, cell clones do not out-compete surrounding cells even when Myc is upregulated due to a scarcity of cellular building blocks (Moreno and Basler, 2004). When we expressed the anti-apoptotic gene p35 on *shmt*-deficient clones, we saw a tendency for an increase in clone number, although this was not statistically significant. However, this could be due to the low number of clones retrieved. Preventing apoptosis in *shmt* mutant cells was not sufficient to rescue clone growth and size, likely as these cells are deficient for the synthesis of several biomolecules, including nucleotides. Considering these findings, we propose that Myc is acting upstream of *shmt* regulating cell division and growth of neuroepithelia (Fig. 6G). Thus, neuroepithelial cells lacking *shmt* when surrounded by WT cells, have a growth disadvantage, suggesting a mechanism similar to ‘cell competition’. We can, however, not exclude that these less fit cells are being eliminated in a cell autonomous manner.

Our data also revealed that the downregulation of Ts in neuroepithelial cells leads to a defective optic lobe formation, similar to that observed in *shmt* mutant brains. This is consistent with a model where the optic lobe defects observed in *shmt* mutants are a consequence of deficient one-carbon metabolism rather than a specific role of Shmt in this tissue. Consistently it has been shown that inhibition of Ts in intestinal stem cells reduces cell proliferation (Sasaki et al., 2021).

As optic lobe neuroepithelia cell number is reduced from early larva in the absence of *shmt*, it is expected that the formation of NBs and their progeny would also be reduced, as indeed observed. A smaller number of neuroepithelial cells generates a smaller number of NBs, which then form a much-reduced number of medulla neurons. As the overall optic lobe morphology is affected by the reduced number of cells in this structure, this is likely responsible for the abnormal localisation of NBs and their daughter medulla neurons. The neuroepithelia morphological defects, as the defects in the formation of the lamina furrow observed during 3rd instar larval stage, could also explain the disorganisation of the optic lobe. During the formation of the lamina furrow, the neuroepithelial cells are required to change their shape to form a deep groove structure (Selleck and Steller, 1991). This furrow is required for the transition from neuroepithelial cells to LPCs and consequently for the formation of lamina neurons. In *shmt* mutants, however, the lamina furrow is not formed nor are the lamina neurons. It remains unclear whether the increased levels of apoptosis in the neuroepithelia of *shmt* mutants contributes to the defects in lamina furrow formation. A lower number of neuroepithelial cells could, for example, loosen the tension in this tissue, hampering the apical constriction of cells likely required for furrow formation. It is interesting to speculate that these defects in the lamina furrow are due to defects in the neuroepithelial cell remodelling in the absence of Shmt. Interestingly it has also been previously shown that the transition from neuroepithelia to medulla NBs is accompanied by the remodelling of the newly formed NSC, which must apically constrict before dividing (Shard et al., 2020). These NSCs at the transition zone, also called epithelial-like neural stem cell (epi-NSC), apically constrict due to myosin pulses and only afterwards undergo an asymmetric cell division to form an NB, which will then generate a GMC and ultimately neurons (Shard et al., 2020). Thus, although we observed that fate determinants are still expressed at the transition zone from neuroepithelia to medulla NBs, the defects in the formation of the medulla could also be due to remodelling defects.

In summary, our results indicate that *shmt* mutant optic lobe morphological defects are a consequence of defective cellular remodelling events, decreased cell fitness and increased levels of apoptosis of the neuroepithelia. The mammalian embryonic neural tube is a similar neuroepithelia tissue that undergoes high proliferation and profound morphological changes requiring apical constriction (Grosse et al., 2016). The neural tube closure is a process that is highly dependent on one-carbon metabolism and on maternal folate status (Engelhardt et al., 2022). In mouse and human embryos with reduced levels of folate, the neural tube fails to close, leading to several neurological defects, such as anencephaly and spina bifida (Grosse et al., 2016; Wilde et al., 2014). Mice lacking Shmt1 (the mammalian cytoplasmatic isoform of Shmt) fail the neural tube closure because of an impaired thymidylate biosynthesis pathway (Anderson and Stover, 2009). Overall, this suggests a conserved role for Shmt and one-carbon metabolism in the regulation of neuroepithelia development.

## MATERIALS AND METHODS

### Fly strains and genetic crosses

RNAi lines used were obtained from the Transgenic RNAi Project (TriP) lines from the Bloomington *Drosophila* Stock Center (BL). RNAi used were UAS-Shmt RNAi (IR) (BL57739) and UAS-Ts IR (BL62237). R31HO9-GAL4 (BL49694) was used as a neuroepithelial cells driver; expression was confirmed with stock UAS-CD8::GFP (our lab). W1118 (a gift from António Jacinto laboratory, NMS, Universidade Nova de Lisboa, Portugal) was used as a WT control, unless stated otherwise.

UAS-Cas9/CyO; R31HO9-GAL4/Tm6b (generated from BL54594 and BL49694) was used as a driver for CRISPR-Cas9 KO in neuroepithelial cells. ShmtTKO.GS04720 (BL81469) was used to induce *shmt* KO.

For MARCM clones generation, the fly strain hsFLP, tubP-Gal80,FRT19A;UAS-CD8::GFP; TubGal4 /Tm6b Tb (our lab), was used as a tool. In addition, the following fly strains were used: Wfrt19Ashmtm3-5/FM7actGFP (generated in this study), UAS-HA-dMyc (a gift from Paola Bellosta laboratory; Bellosta et al., 2005) and UAS-p35 (a gift from Rita Teodoro laboratory, NMS, Universidade Nova de Lisboa, Portugal). The double transgenic fly lines WFRT19Ashmtm3-5/Fm6;UAS-HA-dMyc/CyO and WFRT19Ashmtm3-5/Fm6;UAS-p35/CyO were generated for this study. Yw1118P{neoFRT19A} (BL1744) was used as control.

For the flip-out *shmt* overexpression clones, UAS-Shmt-HA (a gift from L. Miguel Martins; Celardo et al., 2017) was used. Hs^FlpWeak^;;actin>CD2>Gal4UASCD8GFP/Tm6b (our lab) was used as a tool for clone generation. For the rescue experiment the fly strain used was Shmt[+]86F (a gift from Jörg Großhans; Winkler et al., 2017).

### Shmt mutant generation

WFRT19Ashmtm3-5/FM7actGFP was generated by CRISPR-Cas9 as described in Gokcezade et al. (2014). Briefly the guide RNA sequence AGGCCCATGATGCGATCGTG was cloned in a Pu6-Bbsl-chiRNA plasmid (45946, Addgene) and injected directly in FRT19A flies. Hatched flies were crossed to 1st chromosome balancer flies and F1 resulting males were screened for frameshift mutations by the following PCR sequencing primers: Fw_ shmtm3-5: 5′-CTACGGTGGCAACGAGTACA-3′; Rv_ shmtm3-5: 5′-AGAAGATATCGGCCACATCG-3′. The resulting mutant stock was backcrossed to white flies to clean possible second site mutations.

### Fly rearing and dissection

For the RNAi experiments, R31HO9-GAL4 was crossed to UAS candidate RNAi. Fly crosses were reared in yeast-enriched food and kept at 29°C. Brains were dissected at wandering 3rd instar larval stage.

For the *shmt* CRISPR-Cas9 KO experiment, ShmtTKO.GS04720 was crossed with UAS-Cas9/CyO; R31HO9-GAL4/Tm6b and flies and progeny kept at 29°C. Brains were dissected at wandering 3rd instar larval stage.

For most experiments performed to characterise *shmt*^m3-5^ optic lobe phenotype, w-FRT19Ashmtm3-5/FM7actGFP and w1118 fly stocks were reared in food plates for 3 h, after which adult flies were transferred to new plates. Eggs laid during this period were let in the food plates, at 25°C, and brains were dissected at the stated developmental stage of interest: early 2nd (24 h ALH), late 2nd (48 h ALH), early 3rd (60 h ALH), mid 3rd (72 h ALH) and late 3rd (96 h ALH) larval stage.

For apoptosis experiments, w1118 and w-FRT19Ashmtm3-5/FM7actGFP egg-lays were conducted in apple juice plates supplemented with yeast. Newly hatched L1 larvae were collected hourly, transferred to yeast-enriched food and kept at 25°C. Brains were dissected at the developmental stage of interest: early 2nd (24 h ALH), late 2nd (48 h ALH), mid 3rd (72 h ALH) and late 3rd (96 h ALH) larval stage.

For MARCM clones generation, yw1118P{neFRT19A), w-FRT19Ashmtm3-5/FM7actGFP, w-FRT19Ashmtm3-5/Fm6;UAS-HA-dMyc/CyO and w-FRT19Ashmtm3-5/Fm6;UAS-p35/CyO were crossed to hsFlptubGal80FRT19A;;TubGal4UAS-CD8::GFP/Tm6b at 25°C. The larval progeny from these crosses was exposed to 1h 30 heat shocks at 37°C, either 24 h or 48 h ALH. Brains and wing discs were then dissected at wandering 3rd instar larval stage.

For the Shmt overexpression flip-out clones generation, the w1118 and UAS-Shmt-HA stocks were crossed to hs^FlpWeak^;;actin>CD2>Gal4UASCD8GFP/Tm6b and kept at 25°C. The larval progeny was exposed to 1h 30 heat-shock at 37°C, at 48 h ALH. Brains were dissected at wandering 3rd instar larval stage.

The *shmt*^m3-5^ rescue experiment was performed by crossing w-FRT19Ashmtm3-5/FM7actGFP to Shmt[+]86F. Flies were kept at 25°C and brains dissected at wandering 3rd instar larval stage; balancer was excluded by fluorescence and *shmt*^m3-5^ hemizygous male larvae were selected and analysed.

### Antibodies and immunofluorescence

The following primary antibodies were used: rabbit anti-miranda (1:1000, gift from Jürgen Knoblich, Institute of Molecular Biotechnology, Austria); goat anti-deadpan (1:100, AB0356-100, SICGEN); guinea pig anti-deadpan (1:1000, a gift from Rita Sousa Nunes laboratory, King's College London, UK); guinea pig anti-L'sc (1:1200, a gift from Makoto Sato; Suzuki et al., 2013); rabbit anti-shmt (1:1000, a gift from Jörg Großhans; Winkler et al., 2017); mouse anti-Dachshund [1:100, mAbdaC1_1, Developmental Studies Hybridoma Bank (DSHB)]; rat anti-elav (1:50, 7E8A10, DSHB); mouse anti-Armadillo (1:50, N27A1, DSHB); rabbit anti-PH3 (1:1000, 06-570, Millipore); rabbit anti-Dcp1 (1:250, 1679578S, Cell Signaling Technology); DAPI (1:1000, a gift from Alisson Gontijo laboratory, NMS, Universidade Nova de Lisboa, Portugal). The Alexa-conjugated secondary antibodies used were: Alexa Fluor 405, Alexa Fluor 488, Alexa Fluor 568 and Alexa Fluor 647 (1:1000, Invitrogen).

All the immunofluorescence assays were performed as follows unless indicated otherwise: larval brains or wing discs were dissected in 1× PBS solution (pH 7.4), on ice. Sample was fixed in 4% paraformaldehyde (PFA) at room temperature (RT) in a rocker for 30 min; afterwards the tissue was washed three times in PBS with 0.1% Triton X-100 (PBT), blocked with PBS with 0.1% Triton X-100 and 1% normal goat serum (blocking solution) and incubated with the primary antibodies overnight, at 4°C. The following day, the primary antibodies were removed, and the tissue was washed with blocking solution. Secondary antibodies were then added, and let to incubate for 2 h, protected from light, at RT. Afterwards, the sample was washed with PBT and kept in rotation in PBS for an additional 10-20 min. Finally, brains or wing disc were mounted into a slide with a drop of aquapolymount (Polysciences). Immunofluorescent images were acquired on a Carl Zeiss LSM880 microscope and processed using Imaris or ImageJ.

For experiments that included anti-deadpan goat, normal donkey serum was used as a replacement for the blocking solution. For experiments that included anti-armadillo antibody, brains were fixed in 4% PFA with 0.1% of Triton for 30 min and incubated with the primary antibodies for 2 days at 4°C.

For EdU incorporation the Click-It EdU Cell Proliferation Kit for Imaging (C10340, Invitrogen) was used. Larval brains were dissected in 1× PBS solution (pH 7.4) at RT and incubated with EdU at a final concentration of 10µM diluted in PBS for 1 h at RT with rotation. Brains were then immediately fixed in 4% PFA and the protocol of immunofluorescence was followed as described above. After secondary antibody incubation, EdU detection was performed in accordance with the supplier's protocol (30 min, RT, with agitation, protected from light). Brains were then washed and fixed as described above.

### Quantitative PCR

Total RNA was extracted from five wandering 3rd instar larvae using TRIzol reagent (Invitrogen), submitted to DNAse Treatment (TURBO DNA-free kit, AM1907) and then reverse transcripted (Thermo Fisher Scientific RevertAid First Strand Cdna Synthesis Kit, K1622). Quantitative PCR analysis was then performed using GoTaq Qpcr Master Mix (Promega) in an Applied Biosystems QuantStudio5. The sequences of primers used to amplify *shmt* and *actin5a* (endogenous control) were as follows: shmt-F: 5′-GAGTACATCGACCGCATAGAG-3′; shmt-R: 5′-TCCGGAATAAGGCTGCACA-3′; act5c-F: 5′-AGTGGTGGAAGTTTGGAGTG-3′; act5c-R: 5′-GATAATGATGATGGTGTGCAGG-3′.

### Viability assay

The viability assay was performed by crossing w1118×w1118 (control) or w1118×w-FRT19Ashmtm3_5/FM7actGFP (experiment) in apple juice plates. The flies were let to lay eggs for 24 h; eggs developed for 24 h to hatch and give rise to L1 larvae. Fifty WT L1 larvae were transferred to a new apple juice plate, using a brush.

The W1118×w-FRT19Ashmtm3_5/FM7actGFP cross resulted in two genotypes: GFP-positive larvae and GFP-negative larvae. The two genotypes were separated in the L1 phase into two different apple juice plates, 50 larvae per plate.

The larvae that reached the subsequent phase, L2, were counted and then allowed to develop for another 24 h to reach phase L3. All the animals that reached L3 stage were transferred to a vial supplemented with normal food. In this stage of development, the animals were separated by sex. Their subsequent development was quantified and qualified in terms of survival per sex.

### Neuroepithelial cell and neuroblast quantification

For each brain, optic lobe neuroepithelium and NB cell number was the result of the sum of cells counted in three confocal sections. The initial confocal section was selected where NE cells were at their maximum length, with their cell membranes well visible. The following confocal section (middle) was selected at least 5 µm apart from the previous one, to ensure that the same neuroepithelium cell was not counted twice, maintaining the criteria that neuroepithelium cells must be at their maximum length. The same guidelines applied for the final confocal section selected to count NE cells and NBs (Fig. S4A).

### Statistical analyses

Statistical analyses were performed using GraphPad Prism 6. Unpaired two-tailed Student's *t*-test or the Mann–Whitney test was performed to determine statistical significance between two groups. Analysis with more than two groups was conducted with the Bonferroni's multiple comparison test. Clonal frequency in Fig. 6A and Fig. S5A were determined by presence or absence of clones in the neuroepithelia or non-neuroepithelia in the optic lobe region; statistical analysis was carried out using Fisher's exact test.

## Supplementary Material

10.1242/develop.201152_sup1 Supplementary information
