# Supplementary material for: Serine hydroxymethyl transferase is required for optic lobe neuroepithelia development in Drosophila
Source: Development. 2023 Jun 1;150(20):dev201152. doi: 10.1242/dev.201152 (PMC10281515; doi:10.1242/dev.201152)
Supplement: Supplementary information [file develop-150-201152-s1.pdf]

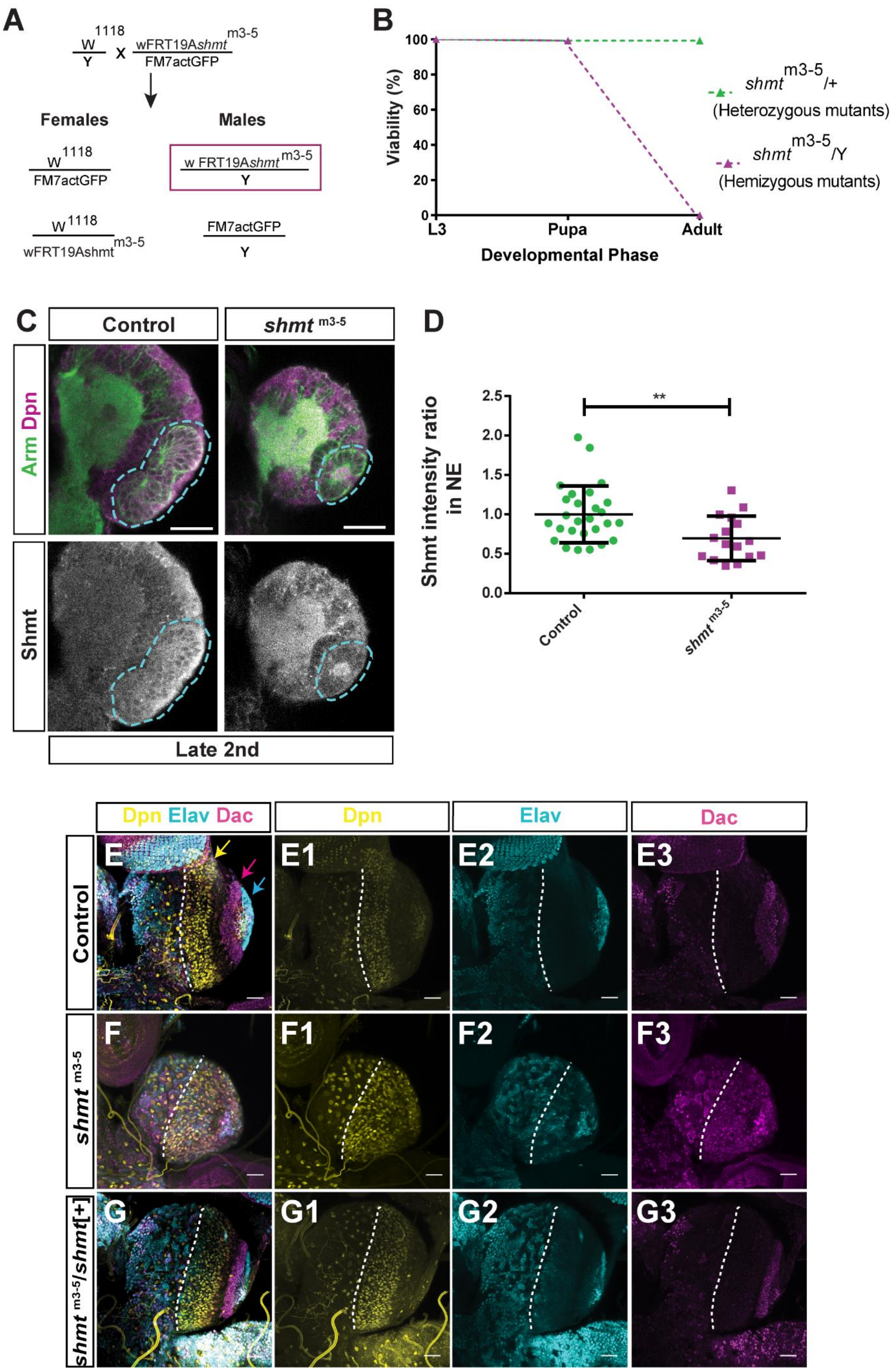

Development • Supplementary information

Fig. S1. Viability analysis of *shmt*$^{m3\text{-}5}$ animals. (A) Possible progeny resulting from the genetic cross used for the viability assay. *shmt*$^{m3\text{-}5}$ parent females were balanced with a GFP positive balancer (Fm7actGFP). For all experiments the progeny that inherits the non-mutated X chromosome (Fm7actGFP) was identified by fluorescence and discarded. The remaining hemizygous males (*wFRT19Ashmt*$^{m3\text{-}5}$*/ y)* and heterozygous female progeny (w^{118}/ *wFRT19Ashmt*$^{m3\text{-}5}$) were separated according to sex in L3 stages and their viability separately tracked. Hemizygous males, *wFRT19Ashmt*$^{m3\text{-}5}$*/ y,* are highlighted with a purple box. **(B)** Survival rates of *shmt*$^{m3\text{-}5}$/+ heterozygous females and *shmt*$^{m3\text{-}5}$/ *y* hemizygous mutant males were measured from 3rd instar larvae to the adult stage. **(C)** Late 2nd Instar larval brains for Control and *shmt*$^{m3\text{-}5}$ stained with anti-Armadillo (Arm, green) to visualize neuroepithelia and anti-Shmt (Shmt, magenta or grey). Dashed lines mark neuroepithelial region. **(D)** Shmt antibody average intensity in NE was quantified and averaged to control. Number of brains: control (n=27), *shmt*$^{m3\text{-}5}$ (n=16). The error bars represent ± standard deviation (SD); **=p<0.01, Mann-Whitney test. **(E-G3)** 3rd instar larval brains of control, *shmt*$^{m3\text{-}5}$ hemizygous mutant and *shmt*$^{m3\text{-}5}$ hemizygous mutant with *shmt*[+] allele, stained with anti-Deadpan (Dpn, yellow) to visualize neuroblasts (NBs), anti-Elav to visualize medulla neurons (Elav, Cyan) and anti-Dachshund (Dac, magenta) to visualize lamina neurons. Dashed outline separates the optic lobe region from the central brain. Yellow arrow indicates the medulla NBs, magenta arrow indicates the lamina neurons, and the cyan arrow indicates the medulla neurons. For all experiments, unless stated otherwise, w1118 was used as control. The scale bar represents 20 µm.

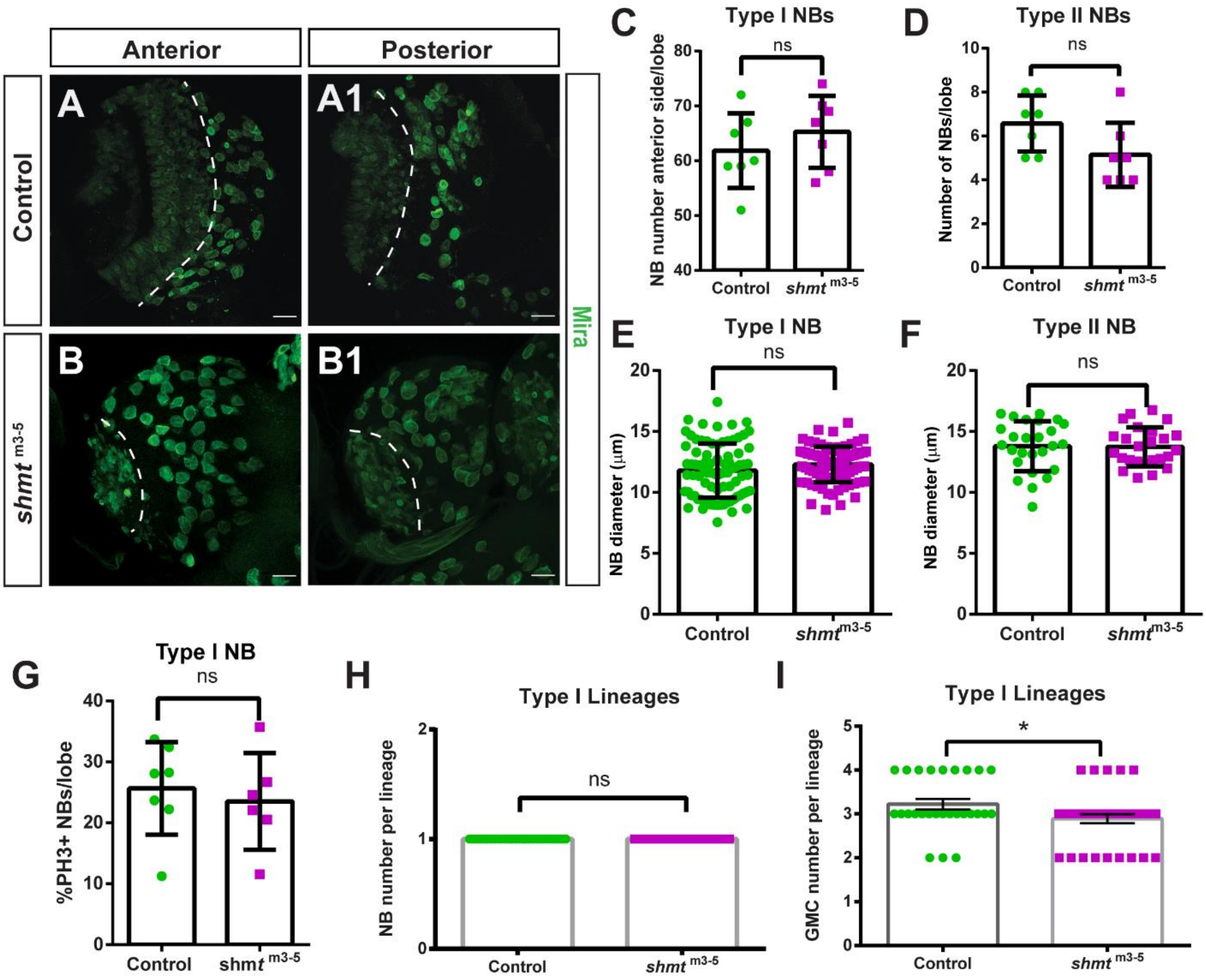

Fig. S2. *shmt* hemizygous mutants have no obvious defects in the central brain. (A-B) Wandering 3rd instar larval brains of control and *shmt*m3-5 stained with anti-Miranda (Mira, green) to visualize neuroblasts (NBs). Dashed outline separates the optic lobe region from the central brain. View from the anterior **(A-B)** and the posterior **(A1-B1)** side of the brain. Scale bars represent 20 µm. **(C)** Quantification of Type I NB number at the anterior side of the brain, for control and *shmt*m3-5. Number of brains quantified: control (n=7), *shmt*m3-5 (n=7). **(D)** Quantification of Type II NB number at the posterior side of the brain, for control and *shmt*m3-5. Number of brains quantified: control (n=7), *shmt*m3-5 (n=7). **(E)** Quantification of Type I NBs size at the anterior side of the brain, for control and *shmt*m3-5. Number of brains quantified: control (n=7), *shmt*m3-5 (n=8). **(F)** Quantification of Type II NB size in the posterior side of the brain, for control and *shmt*m3-5. Number of brains quantified: control (n=5), *shmt*m3-5 (n=8). **(G)** Quantification of PH3+ Type I NBs, for control and *shmt*m3-5. Number of brains quantified: control (n=7), *shmt*m3-5 (n=6). **(H)** Quantification of Type I NB number per Type I NB lineage, for control and *shmt*m3-5. Number of brains quantified: control (n=5), *shmt*m3-5 (n=5). **(I)** Quantification of ganglion mother cells (GMCs) per Type I NB lineage, for control and *shmt*m3-5. Number of brains quantified: control (n=5), *shmt*m3-5 (n=5). For all experiments, w1118 was used as control. The error bars represent ± standard deviation (SD); *=p<0.05; ns = not significant, Mann-Whitney test.

Development • Supplementary information

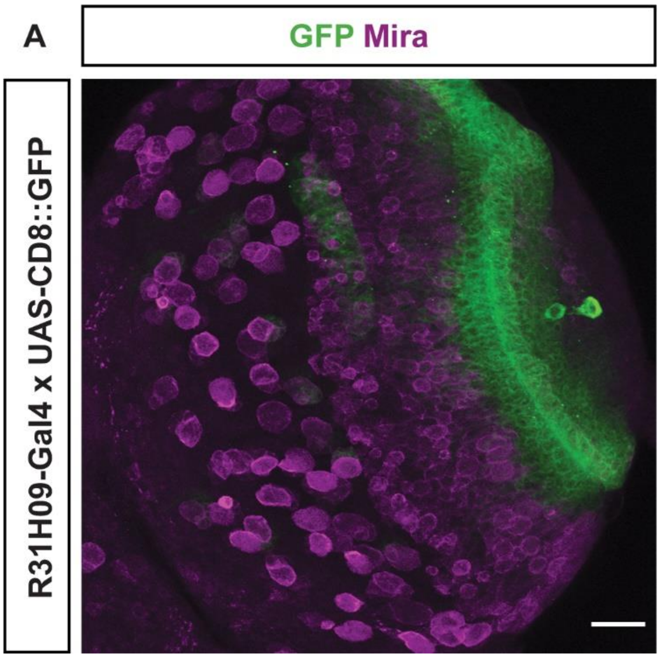

Fig. S3. Neuroepithelium R31H09 driver expression in larva. (A) Wandering 3rd instar larval brain of R31H09-Gal4, driving expression of UAS-CD8::GFP. Anti-Miranda (Mira, magenta) was used to visualize neuroblasts (NBs) and CD8::GFP (GFP, green) to visualize expression pattern. Scale bar represents 20 µm.

Development • Supplementary information

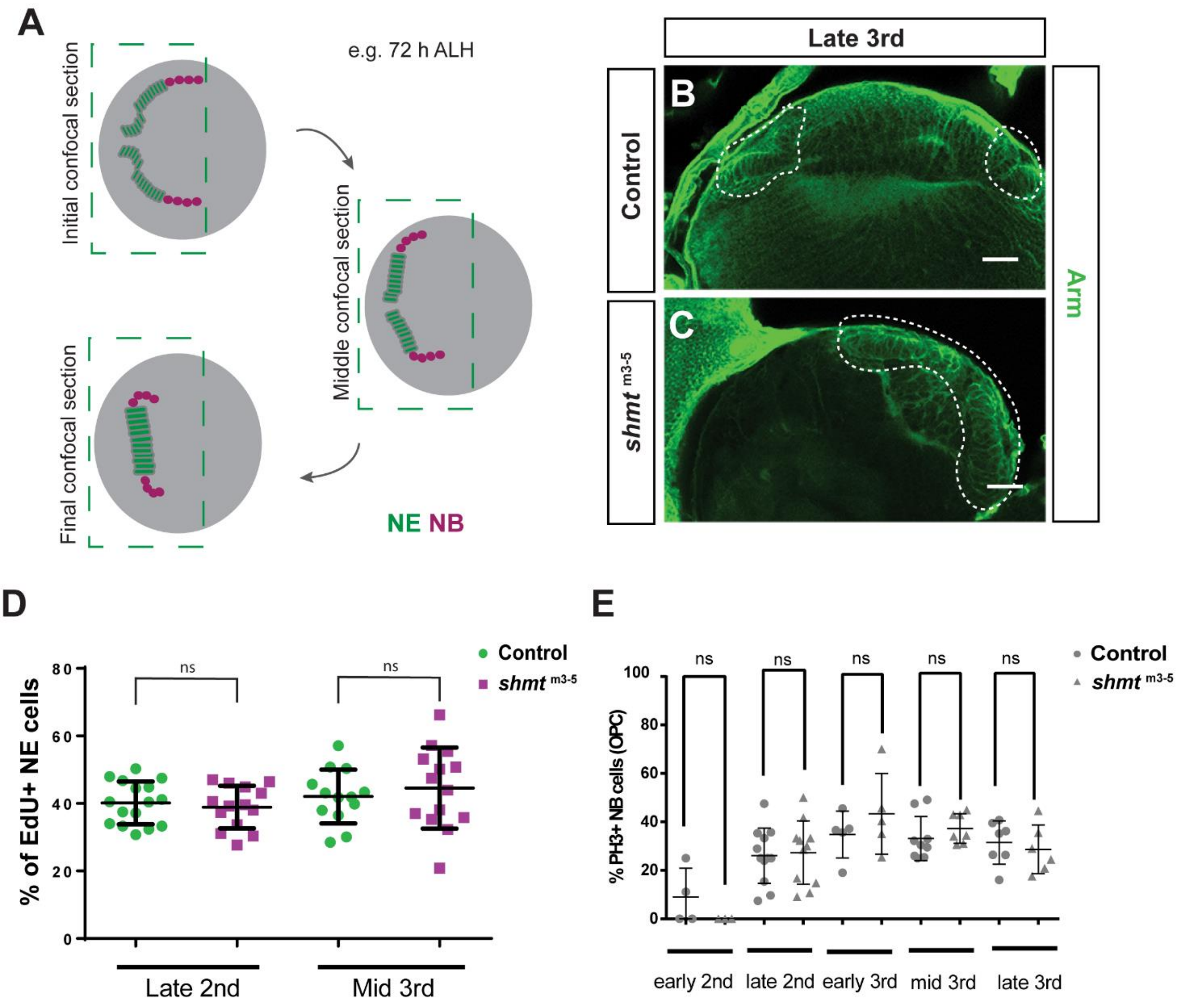

Fig. S4. Loss of *shmt* leads to smaller neuroepithelia with morphological defects but with no alterations in mitotic rates. (A) Diagram exemplifying how neuroepithelial cell (NE, green) and neuroblast (NB, magenta) number was counted. NE and NB number was the result of the sum of the cells counted in 3 confocal sections per brain. The initial confocal section was selected where NE cells were at their maximum length, with their cell membranes well visible. The following confocal section (middle) was selected at least 5µm apart from the prior, to ensure that the same neuroepithelium cell was not counted twice, maintaining the criteria that NE cells have to be at their maximum length. Same guidelines appled for the final confocal section selected to count NE cells and NBs. **(B-C)** Larger view of images in figure 4F1 and 4K1. Late 3rd instar larval brains (96h after larval hatching, ALH) stained with anti-Armadillo (Arm, green) to visualize neuroepithelia, for control and *shmt*$^{m3\text{-}5}$. Dashed lines mark neuroepithelial region. Scale bars represent 20 µm. **(D)** Percentage of EdU+ cells in neuroepithelium for control and *shmt*$^{m3\text{-}5}$, at late 2nd and mid 3rd Instar larvae. Number of brains quantified for control: late 2nd (48h ALH, n=16), mid 3rd (72h ALH, n=13). Number of brains quantified for *shmt*$^{m3\text{-}5}$: late 2nd (48h ALH, n=14), mid

Development • Supplementary information

3rd (72h ALH, n=14). **(E)** Quantification of PH3+ labelled OPC neuroblasts (NBs) for control and *shmt*$^{m3\text{-}5}$ during larval development. Number of brains quantified for control: early 2nd (24h ALH, n=6), late 2nd (n=12), early 3rd (60h ALH, n=5), mid 3rd (n=9), late 3rd (n=7). Number of brains quantified for *shmt*$^{m3\text{-}5}$: early 2nd (n=6), late 2nd (n=11), early 3rd (n=5), mid 3rd (n=7), late 3rd (n=6). For all experiments, w1118 was used as control. The error bars represent ± standard deviation (SD); ns = not significant; Bonferroni's multiple comparison test.

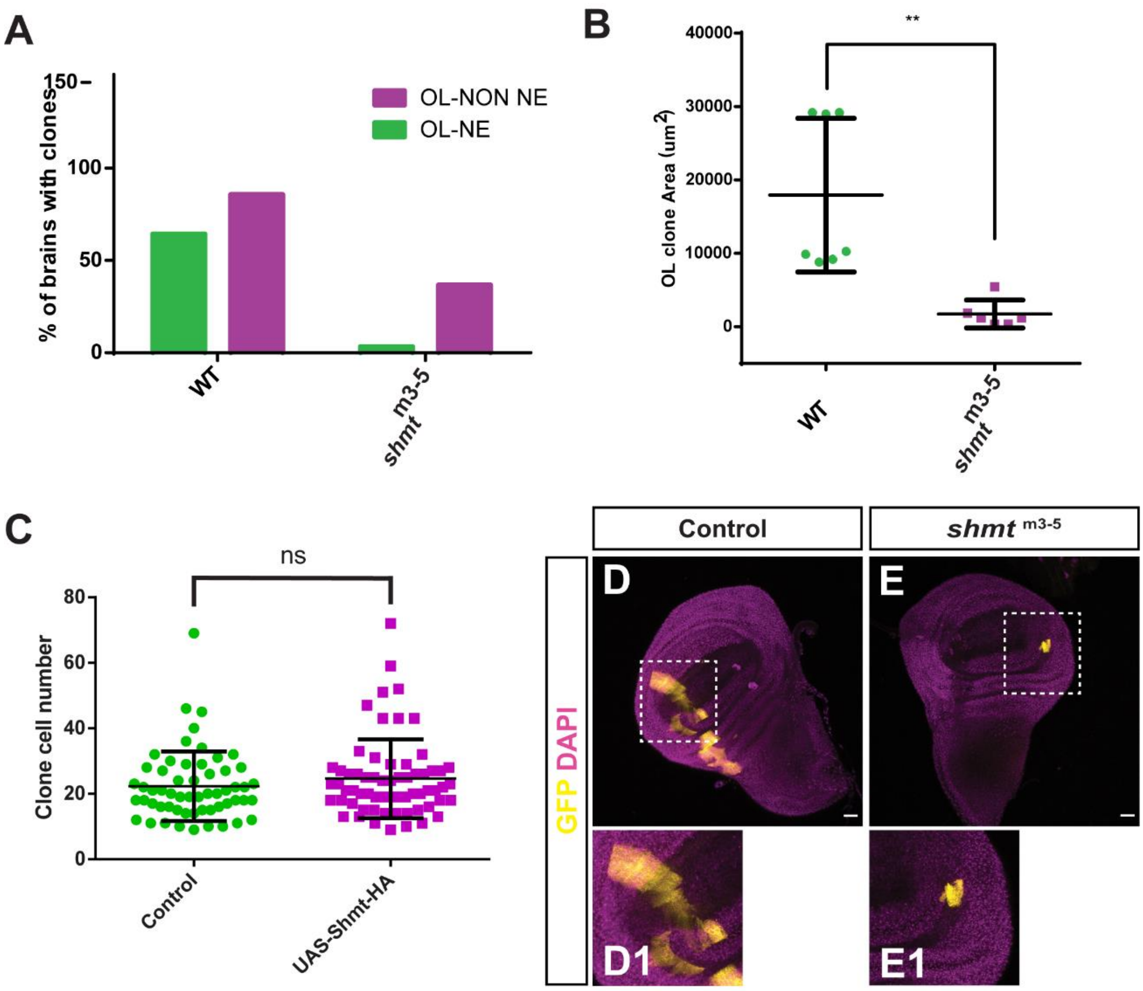

Fig. S5. *shmt* neuroepithelial clones are rare and have a growth disadvantage. (A) Quantification of brains with optic lobe clones in NE (OL NE) and brains with optic lobe clones not localized on NE (OL-NON NE) of control and *shmt*$^{m3-5}$, induced at 24h ALH. Number of brains: WT (n=14), *shmt*$^{m3-5}$ (n=29). Statistical analysis was done with Fisher´s exact test. OL-NE (clones present vs. absent): WT vs. *shmt*$^{m3-5}$ p-value <0.0001. OL- NON-NE (clones present vs. absent): WT vs. *shmt*$^{m3-5}$ ns= not significant. **(B)** Quantification of the OL (OL NE+ OL-NON NE) clonal area for control and *shmt*$^{m3-5}$, induced at 24h ALH. Number of clones: WT (n=7), *shmt*$^{m3-5}$ (n=6). **(C)** Quantification of cell number per individual control or UAS-Shmt-HA flip-out clone in the optic lobe. Number of clones: control (n=57); UAS-Shmt-HA (n=64) The error bars represent ± standard deviation (SD); **=p<0.01; ns = not significant; Mann-Whitney test. **(D-E)** Wandering 3rd instar larval wing discs with control or *shmt*$^{m3-5}$ epithelium MARCM clones. The white dashed outlines indicate the region where the close up was done. Larval wing disc were stained with anti-DAPI (DAPI, magenta) and clones marked by CD8::GFP (GFP, yellow). (**D1-E1)** Close up of clone's area of the indicated genotype. All MARCM experiments used FRT19A as control; for flip-out clones, w1118 was used as control. Scale bars represent 20 µm.

Development • Supplementary information
